# PTEN deficiency promotes macrophage infiltration and hypersensitivity of prostate cancer to IAP antagonist/radiation combination therapy

**DOI:** 10.18632/oncotarget.6955

**Published:** 2016-01-20

**Authors:** Chris W.D. Armstrong, Pamela J. Maxwell, Chee Wee Ong, Kelly M. Redmond, Christopher McCann, Jessica Neisen, George A. Ward, Gianni Chessari, Christopher Johnson, Nyree T. Crawford, Melissa J. LaBonte, Kevin M. Prise, Tracy Robson, Manuel Salto-Tellez, Daniel B. Longley, David J.J. Waugh

**Affiliations:** ^1^ Movember Centre of Excellence, Centre for Cancer Research and Cell Biology, Queen's University Belfast, Belfast, Northern Ireland; ^2^ School of Pharmacy, Queen's University Belfast, Belfast, Northern Ireland; ^3^ Astex Pharmaceuticals, Cambridge, UK

**Keywords:** prostate cancer, PTEN, radiation, microenvironment, IAP

## Abstract

PTEN loss is prognostic for patient relapse post-radiotherapy in prostate cancer (CaP). Infiltration of tumor-associated macrophages (TAMs) is associated with reduced disease-free survival following radical prostatectomy. However, the association between PTEN loss, TAM infiltration and radiotherapy response of CaP cells remains to be evaluated. Immunohistochemical and molecular analysis of surgically-resected Gleason 7 tumors confirmed that PTEN loss correlated with increased CXCL8 expression and macrophage infiltration. However PTEN status had no discernable correlation with expression of other inflammatory markers by CaP cells, including TNF-α. *In vitro*, exposure to conditioned media harvested from irradiated PTEN null CaP cells induced chemotaxis of macrophage-like THP-1 cells, a response partially attenuated by CXCL8 inhibition. Co-culture with THP-1 cells resulted in a modest reduction in the radio-sensitivity of DU145 cells. Cytokine profiling revealed constitutive secretion of TNF-α from CaP cells irrespective of PTEN status and IR-induced TNF-α secretion from THP-1 cells. THP-1-derived TNF-α increased NFκB pro-survival activity and elevated expression of anti-apoptotic proteins including cellular inhibitor of apoptosis protein-1 (cIAP-1) in CaP cells, which could be attenuated by pre-treatment with a TNF-α neutralizing antibody. Treatment with a novel IAP antagonist, AT-IAP, decreased basal and TNF-α-induced cIAP-1 expression in CaP cells, switched TNF-α signaling from pro-survival to pro-apoptotic and increased radiation sensitivity of CaP cells in co-culture with THP-1 cells. We conclude that targeting cIAP-1 can overcome apoptosis resistance of CaP cells and is an ideal approach to exploit high TNF-α signals within the TAM-rich microenvironment of PTEN-deficient CaP cells to enhance response to radiotherapy.

## INTRODUCTION

Radiotherapy (RT) constitutes a major treatment modality used in patients presenting with localized prostate cancer (CaP) [1]. Despite RT and surgery being curative in up to 50% of patients, approximately one third of men will exhibit biochemical recurrence and disease progression towards fatal castrate-resistant prostate cancer (CRPC) [2, 3]. Loss of the haplo-insufficient tumor suppressor PTEN has recently been identified as a prognostic factor for patient relapse following RT [4]. Furthermore, we have previously identified the chemokine CXCL8 as a mediator of PTEN-deficient tumorigenesis [5].

The revised “Hallmarks of Cancer” emphasizes the importance of the tumor microenvironment in tumorigenesis [6]. An increasing number of reports also highlight the impact of microenvironment-derived inflammatory cytokine signaling or stromal-derived pro-angiogenic signals in driving radioresistance [7, 8]. Surprisingly, knowledge of microenvironment-mediated radioresistance in CaP is poorly characterized but given the strong association of inflammation with the disease, there is a high potential that the immune cell infiltrate may modulate the overall tumor response to RT in early-stage disease. Tumor-associated macrophages (TAM) have been identified as a significant component of the inflammatory cell infiltrate in CaP and may influence disease progression through their ability to release soluble signaling factors within the tumor boundaries [9, 10]. TAM infiltration has previously been correlated with disease free survival (DFS) following radical prostatectomy (RP) (11–13). However, no such studies have been reported regarding RT response.

TNF-α is a cytokine primarily produced by macrophages and activates the extrinsic apoptosis pathway following its binding to cell surface death receptors on tumor cells [11, 12]. CaP cells are notoriously resistant to the action of TNF-α, and rather than promoting apoptosis, TNF-α instead preferentially promotes the activation of NFκB-dependent pro-survival signaling [13, 14]. This switch from death-promoting to survival signaling is regulated by the expression and function of the cellular inhibitor of apoptosis protein-1 and -2 (cIAP-1 and cIAP-2) (18). Overexpression of cIAP-1 may therefore underpin resistance of CaP cells to TNF-α. In addition, therapeutic targeting of cIAP-1 may serve as a potent strategy to sensitize CaP cells to TNF-α mediated cell death.

Under normal conditions, cIAP-1 expression is tightly regulated by the action of mitochondrial protein Smac. Loss of mitochondrial membrane permeability results in release of Smac into the cytosol where it can induce proteosomal degradation of cIAP-1 and cIAP-2, whilst directly inhibiting XIAP [15, 16]. Dysfunctional Smac-mediated regulation of apoptosis is a common feature in malignant disease and has resulted in the development of a novel class of drugs known as Smac-mimetics [17, 18]. AT-IAP is a dual cIAP-1 and XIAP antagonist which has demonstrated pre-clinical efficacy, in combination with the histone deacetylase inhibitor vorinostat, in models of mesothelioma [19].

This study aimed to determine the impact of TAM infiltration, and specifically the effects of treatment-induced TNFα signaling in modulating RT response of CaP cells. We show that administration of the c-IAP/x-IAP antagonist, AT-IAP, directs a pro-apoptotic response to TNF-α signaling, increasing the sensitivity of prostate cancer cells to RT. Furthermore, this raises the potential to utilize radiotherapy beyond the localized stage of disease.

## RESULTS

### Depleted PTEN expression in patient samples correlates with increased CXCL8 expression

**Figure 1:**
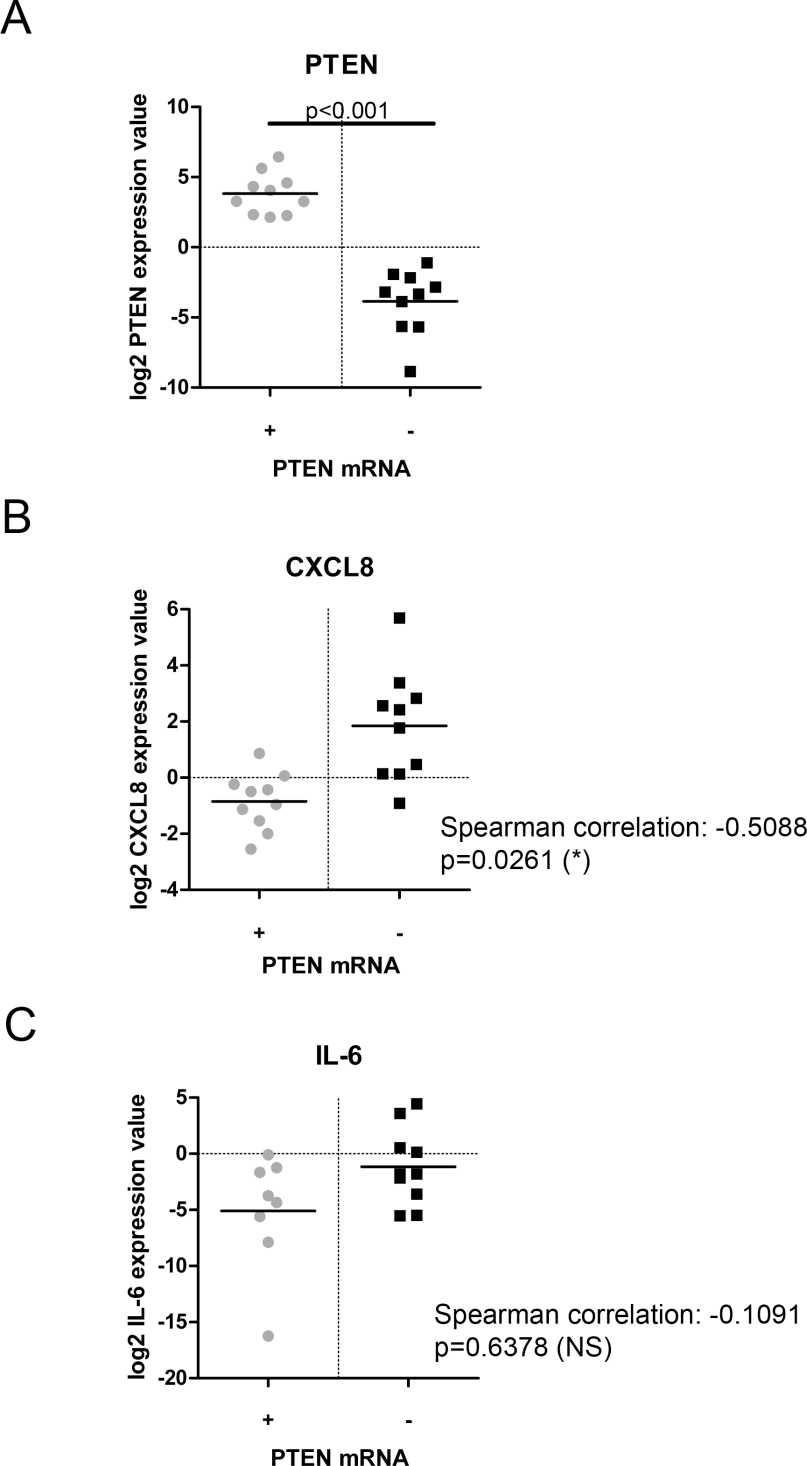
Comparative analysis of PTEN-status and cytokine expression in prostate cancer patient samples (**A**) Scatter plot showing validation of PTEN-status profiling in prostate cancer patient samples. The data presented confirms loss of PTEN mRNA expression following cohort separation by RT-PCR. (**B**) Scatter plot showing CXCL8 gene expression in prostate cancer patient samples separated by PTEN mRNA status. (**C**) Scatter plot showing IL-6 gene expression in prostate cancer patient samples separated by PTEN mRNA status. Statistically significant differences were determined using the Spearman correlation protocol (*p < 0.05; **p < 0.01; ***p < 0.001).

We have previously proposed that augmentation of CXCL8 signaling is a crucial mediator of PTEN-depleted tumorigenesis in CaP [5]. To verify our prior *in vitro* cell-line-derived data, we conducted a molecular analysis on 28 surgically-resected Gleason 7 tumors to establish the correlation of PTEN status against CXCL8 gene expression. Molecular profiling by high throughput gene expression discriminated these samples into two cohorts, stratified on either high or low PTEN expression. Validation experiments confirmed separation of these distinct PTEN populations using RT-PCR analysis of mRNA expression (*p* < 0.001; Figure 1A). Cytokine profiling of these samples confirmed that low PTEN mRNA expression was correlative with increased CXCL8 mRNA (Spearman correlation: −0.5088; *p* = 0.0261) (Figure 1B). Conversely, subsequent profiling of these macro-dissected tumor samples confirmed that PTEN status did not correlate with significant changes in intrinsic expression of other cytokines including IL-6 (Spearman correlation: −0.1091; *p* = 0.6378) (Figure 1C). Other cytokines analyzed included CXCL1, CXCL2 and CXCL5 (data not shown).

### CXCL8 induces chemotaxis of radioresistance-promoting THP1's in a PTEN-deficient setting

**Figure 2:**
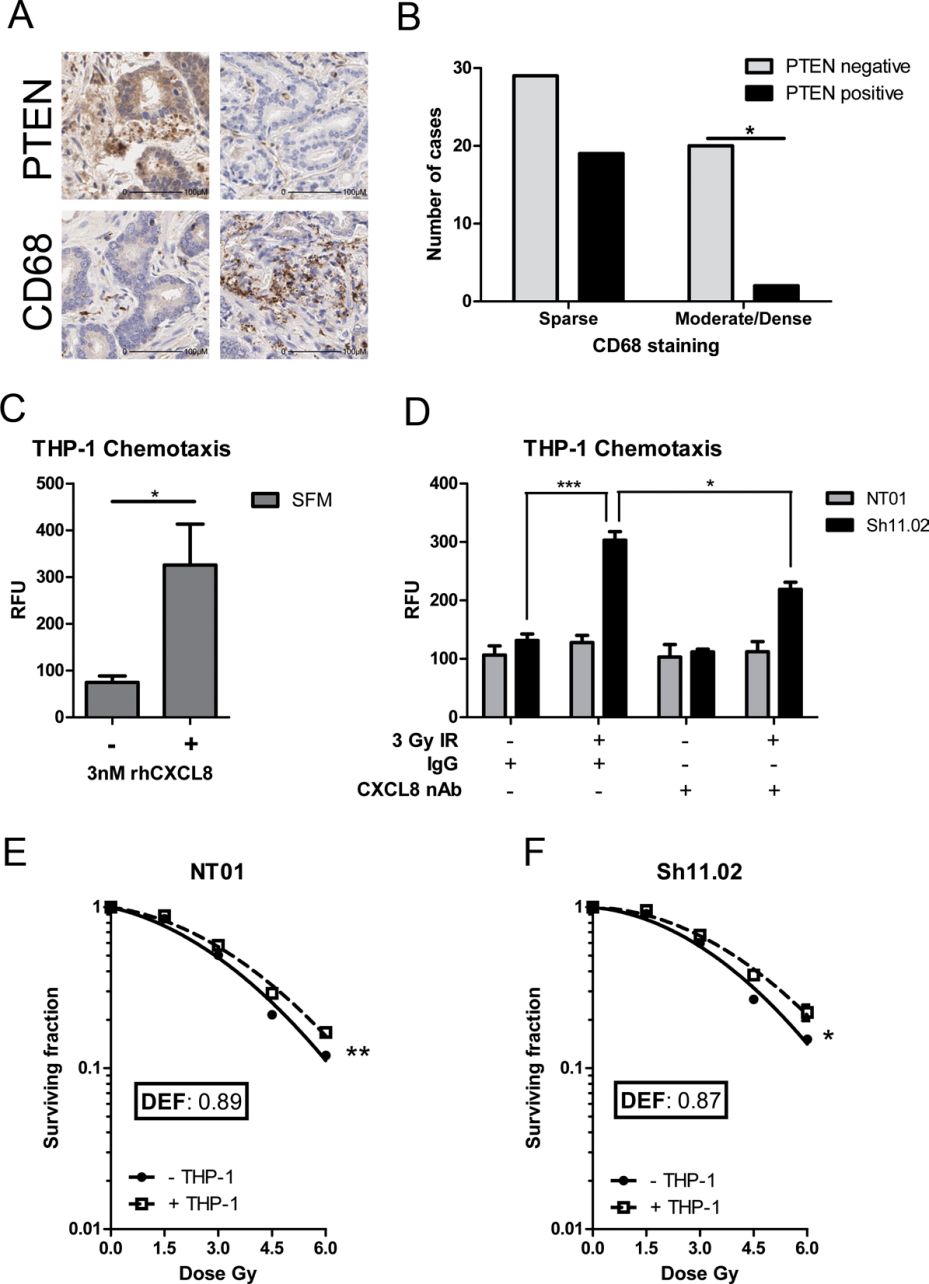
CXCL8 induces chemotaxis of radioresistance-promoting macrophages in a PTEN-deficient setting (**A**) Immunohistochemical staining of PTEN and CD68 in a prostate tissue microarray (*n* = 70). Presented images representative of results across all cases. (**B**) Bar graph demonstrating the correlation between PTEN status and CD68. Statistical analysis was performed using a Chi-squared test; *p* = 0.011. (**C**) Bar graph showing the effect of 3 nM CXCL8 in modulating cell migration of THP-1 cells. (**D**) Bar graph demonstrating the effect of conditioned serum-free media from irradiated PTEN-expressing DU145 NT01 and PTEN-depleted DU145 Sh11.02 cells on THP-1 cell migration. The addition of a CXCL8 neutralizing antibody represses IR-induced cell migration. Clonogenic survival curves showing the effect of THP-1 co-culture on the radiation response of (**E**) NT01 and (**F**) Sh11.02 cells. Data shown is the mean plus or minus standard error of the mean value, calculated from a minimum of three independent experiments. Statistically significant differences were determined by performing a two-tailed Students *t*-test for migration experiments or two-way ANOVA for clonogenic assays (*p < 0.05; **p < 0.01; ***p < 0.001).

CXCL8 was initially characterized as a potent chemoattractant for leukocyte-derived immune cells [22]. Given the up-regulation of CXCL8 expression detected in PTEN-deficient tumors, further IHC analysis was performed to characterize the levels of CD68-positive macrophages detected within prostate patient samples (Figure 2A). Moderate to dense infiltration of CD68-positive macrophages was correlated with loss of PTEN protein expression across 70 analyzable cases (*p* < 0.05). Lower levels of macrophage infiltration were detected within PTEN-positive tumors (Figure 2B). *In vitro* assays confirmed the function of CXCL8 in potentiating chemotactic migration of THP-1 cells, used in this context as a representative macrophage-like cell [243 ± 66% above baseline migration towards serum-free medium; (Figure 2C)]. Furthermore, our assays demonstrated that the conditioned media (CM) harvested from irradiated PTEN-deficient Sh11.02 cells was capable of inducing THP-1 chemotaxis, and that this response could be partially inhibited following pre-treatment with a CXCL8 neutralizing antibody; however, this effect was not observed in PTEN-expressing NT01 cells (Figure 2D). Irradiating CaP cells can induce release of a multitude of cytokines (data not shown) and therefore total inhibition of cell migration may not be possible without considering this extensive signaling network.

To establish how macrophage infiltration may affect therapeutic response, colony formation assays were used to characterize radiation sensitivity of PTEN-modulated DU145 cells, cultured in the presence or absence of THP-1 cells. Co-culture with THP-1 cells increased the resistance of both PTEN-positive NT01 and PTEN-deficient Sh11.02 cells to ionizing radiation (IR), with calculated dose enhancement factors (DEF) of 0.89 and 0.87, respectively. The equivalent degree of sensitization observed suggests that the mechanism of macrophage-afforded resistance was independent of the intrinsic PTEN status of the CaP cells (Figure 2E and 2F). These experiments were repeated in the PTEN-null PC3 cell line and although showing a trend towards monocyte-driven radioresistance at the higher dose points, no significant difference in radiosensitivity was observed (Supplementary Figure 1A). This was also the case when PTEN expression was reconstituted under the control of a tetracycline-inducible promoter (Supplementary Figure 1B).

### IR induces secretion of TNF-α from THP-1 cells but not from CaP cells

**Figure 3:**
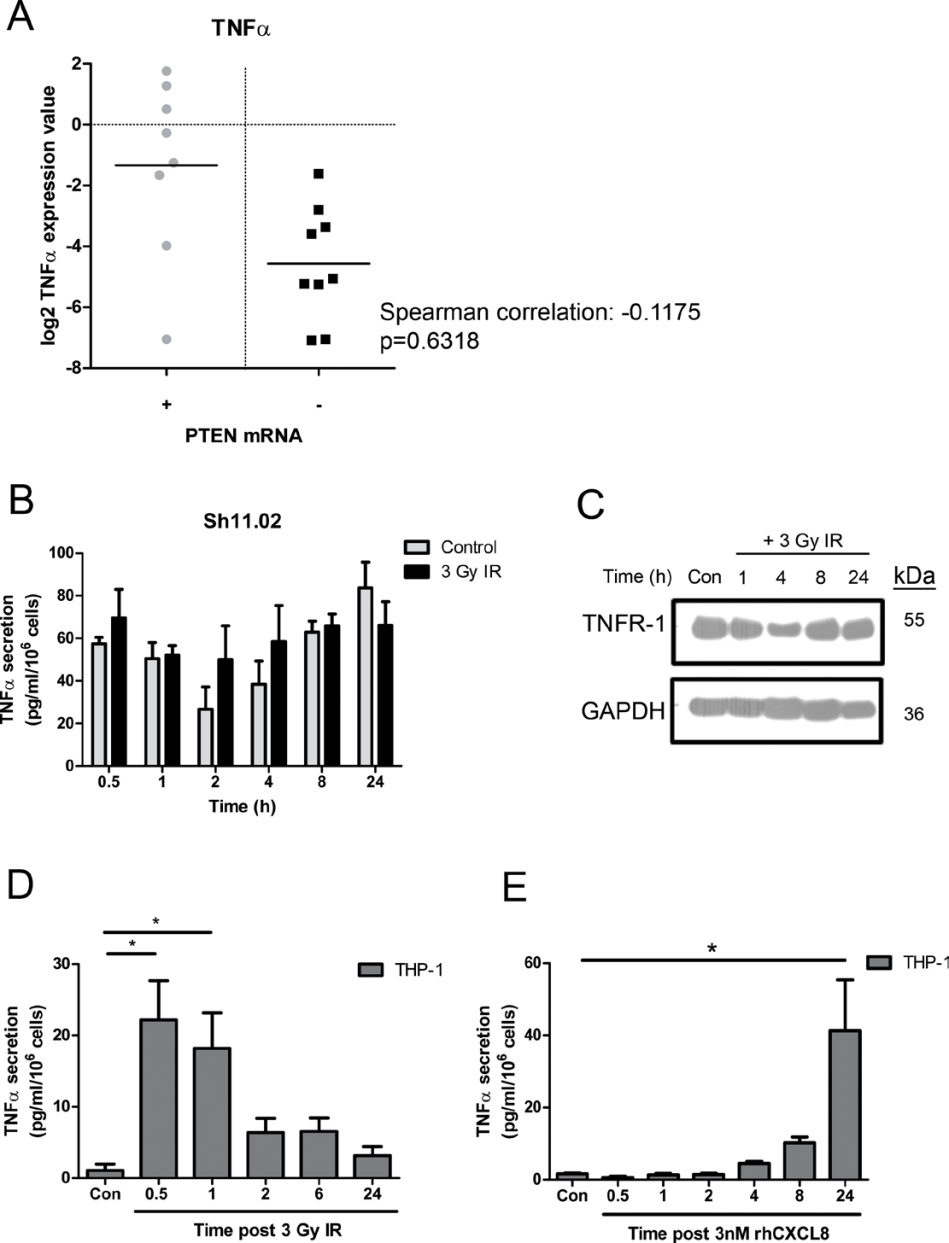
Impact of ionizing radiation and PTEN-status on TNF-α signaling in prostate cancer and THP-1 cell lines (**A**) Scatter plot illustrating TNF-α gene expression in prostate cancer patient samples classified by PTEN mRNA levels. (**B**) Bar graph showing TNF-α secretion from PTEN-depleted Sh11.02 cells following treatment with a single 3 Gy dose of IR. (**C**) Immunoblot demonstrating the impact of 3 Gy IR on TNFR-1 expression in Sh11.02 cells. (**D**) Bar graph showing TNF-α secretion levels from THP-1 cells following treatment with 3 Gy IR. (**E**) Bar graph showing TNF-α secretion levels from THP-1 cells following treatment with 3 nM CXCL8. Data shown is the mean plus or minus standard error of the mean value, calculated from a minimum of three independent experiments. Statistically significant differences were determined by performing a two-tailed Students *t*-test (*p < 0.05; **p < 0.01; ***p < 0.001).

Alongside CXCL8, the cytokine TNF-α has recently been implicated with reduced overall survival and time to castration resistance in CaP patients [23]. Therefore the linkage of PTEN status to TNF-α expression in malignant prostate cells was also determined. Analysis of DU145 NT01 and Sh11.02 cells indicated that loss of PTEN had no effect on TNF-α secretion or mRNA levels in these isogenic lines (Supplementary Figure 2A and 2B). Furthermore, analysis of patient samples confirmed no correlation between PTEN mRNA levels and TNF-α gene expression (Spearman correlation: −0.1175; *p* = 0.6318) (Figure 3A). Since we observed no intrinsic correlation of PTEN with TNF-α expression, experimental results shown are derived from analysis of the PTEN-deficient Sh11.02 cell line while corresponding results derived from PTEN-positive NT01 cells presented in Supplementary Figures 2, 3 and 4.

The effect of radiation exposure upon TNF-α secretion in CaP cells was also determined. Treatment of PTEN-deficient Sh11.02 cells with 3 Gy IR did not augment TNF-α secretion levels compared to time-matched control samples, ranging from between 20–80 pg/ml/10^6^ cells across a 24 h time course (Figure 3B). Ionizing radiation also had no effect on TNF-α mRNA or protein expression (Supplementary Figure 2) or on expression of tumor necrosis factor receptor-1 (TNFR-1) in these cells (Figure 3C). Consistent with the secretion of TNF-α and capacity of DU145 Sh11.02 cells and DU145 NT01 cells to respond in an autocrine manner, addition of 10 ng/ml recombinant-human TNF-α (rh-TNF-α) had a minimal impact on radiation sensitivity as determined in clonogenic assays (Supplementary Figure 5).

Macrophages are a rich source of pro-inflammatory TNF-α. Justified by the enrichment of CD68-positive macrophages detected by IHC in PTEN-deficient prostate tumors, we assessed how exposure to IR modulated TNF-α secretion from THP-1 cells. Treatment with 3 Gy IR promoted a rapid increase in the level of TNF-α secretion observed, with a 21.09 ± 5.5 fold increase evident within 30 min of exposure (Figure 3D). In addition, we also characterized whether the secretion of CXCL8 from PTEN-deficient tumor cells may affect the level of macrophage-derived TNF-α secretion. Treatment with recombinant human-CXCL8 (rh-CXCL8; 3 nM) was shown to induce the secretion of TNF-α from THP-1's, though secretion levels were shown to peak as a consequence of prolonged stimulation (Figure 3E). Thus the release of CXCL8 from the PTEN-deficient tumor cells may also potentiate the IR-induced, macrophage-derived elevation of TNF-α signaling within the microenvironment of PTEN-deficient tumors.

### IR-induced TNF-α from THP-1's promotes NFκB-mediated pro-survival signaling in CaP cells

**Figure 4:**
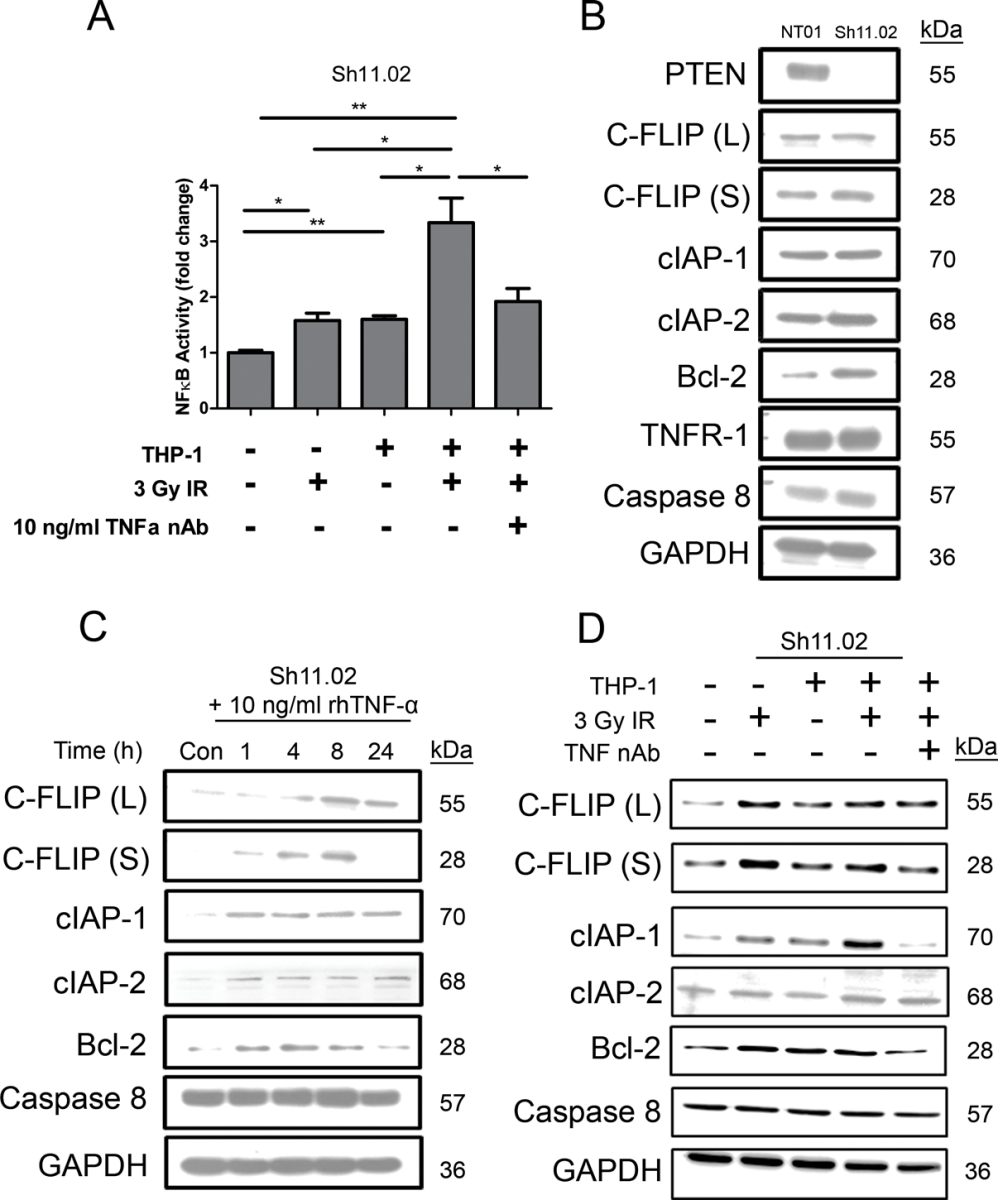
Impact of radiation-induced THP-1 derived TNF-α on NFκB pro-survival signaling (**A**) Bar graph illustrating luciferase reporter assay analysis of NFκB activity in Sh11.02 cells. Different experimental conditions included THP-1 co-culture, exposure to 3 Gy IR and treatment with a TNF-α neutralizing antibody (10 ng/ml). (**B**) Immunoblot showing basal expression of NFκB-regulated anti-apoptotic targets in PTEN-modulated DU145 populations. (**C**) Immunoblot showing the effect of 10 ng/ml recombinant TNF-α treatment on expression of NFκB-regulated anti-apoptotic targets in Sh11.02 cells. (**D**) Immunoblot illustrating expression of NFκB targets in Sh11.02 cells 4 h following 3 Gy IR in the presence or absence of THP-1 co-culture and TNF-α neutralizing antibody. Data shown is the mean plus or minus standard error of the mean value, calculated from a minimum of three independent experiments. Statistically significant differences in luciferase assay results were determined by performing a two-tailed Students *t*-test (*p < 0.05; **p < 0.01; ***p < 0.001).

Experiments were conducted to evaluate how addition of THP-1 cells affected the degree of NFκB activation observed in DU145 Sh11.02 cells, in the absence and presence of radiation. Co-culture with THP-1 cells alone increased the level of NFκB activity within Sh11.02 cells by 60.1 ± 7.8% (*p* < 0.01). However, irradiation of the DU145 Sh11.02/THP-1 co-culture model promoted a 233.6 ± 44.3% increase in NFκB activity, as detected by luciferase reporter assays (*p* < 0.01). The increase in NFκB-driven luciferase activity was attenuated but not abrogated in the presence of a TNF-α neutralizing antibody (*p* < 0.05; Figure 4A). Similar results were observed in the PTEN-expressing DU145 NT01 cell line (Supplementary Figure 3B).

The effect of NFκB activation on the expression of downstream pro-survival effectors was evaluated by immunoblotting. Of the proteins analyzed, only basal expression of Bcl-2 was different between unstimulated PTEN-expressing NT01 and PTEN-deficient Sh11.02 cells (Figure 4B). To model the effects of radiation-induced TNF-α secretion from THP-1 cells, we stimulated Sh11.02 cells with a 10 ng/ml bolus of rhTNF-α over a 24 h time course. Analysis of Sh11.02 protein lysates identified that rhTNF-α increased the expression of several anti-apoptotic proteins including c-FLIP, cIAP-1, cIAP-2 and Bcl-2, whilst having no effect on levels of caspase 8 (Figure 4C). Furthermore, exposure of our Sh11.02/THP-1 co-culture system to 3 Gy IR further elevated the expression of c-FLIP (L) and noticeably increased cIAP-1 levels after 4 h (Figure 4D). Interestingly, this increase in cIAP-1 expression could be prevented by pre-treating with a TNF-α neutralizing antibody.

Given the coupling of TNF-α signaling to pro-survival protein expression, we conducted colony count assays to determine whether repression of IR-induced, macrophage-derived TNF-α signaling could influence cell survival. Administration of a neutralizing antibody to TNF-α failed to decrease cell survival at clinically-relevant doses of IR in either the DU145 NT01 or Sh11.02 cells (Supplementary Figure 5B and 5D respectively). However, a degree of enhancement was seen to high dose IR at levels which are currently under investigation in hypo-fractionation protocols.

**Figure 5:**
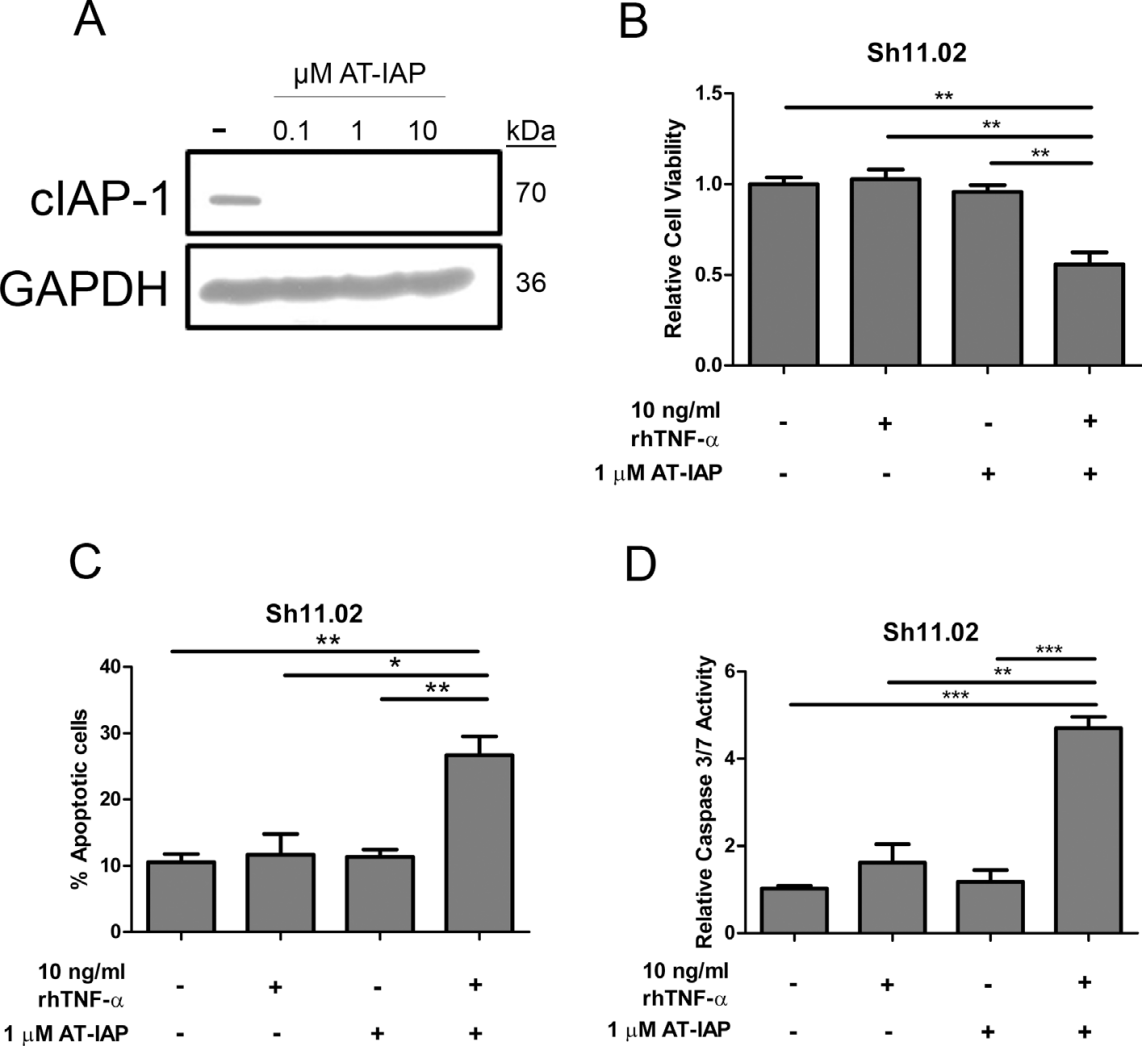
Impact of AT-IAP on cIAP-1 expression and DU145 Sh11.02 cell viability (**A**) Immunoblot showing cIAP-1 expression in Sh11.02 cells following treatment with 0.1, 1 or 10 μM AT-IAP. Equal protein loading was confirmed by re-probing for GAPDH. (**B**) Bar graph presenting MTT assay analysis of Sh11.02 cells 72 h following treatment with 10 ng/ml rhTNF-α, 1 μM AT-IAP, or a combination of both. (**C**) Bar graph illustrating flow cytometry data following Annexin V/PI staining of Sh11.02 cells. Different treatment conditions were similar to those mentioned above. (**D**) Bar graph showing caspase 3/7 activity of Sh11.02 cells following treatment with rhTNF-α, AT-IAP or both in combination for 24 h. Data shown is the mean plus or minus standard error of the mean value, calculated from a minimum of three independent experiments. Statistically significant differences were determined by performing a two-tailed Students *t*-test (*p < 0.05; **p < 0.01; ***p < 0.001).

### AT-IAP induces CaP cell apoptosis through inhibition of cIAP-1

Determining that TNF-α signaling preferentially induced a NFκB pro-survival phenotype raised the potential of targeting upregulated transcriptional targets of this pathway. One of these anti-apoptotic regulators was cIAP1; a protein which is normally under tight regulation via the ability of mitochondrial Smac to induce proteosomal degradation. AT-IAP is a novel dual antagonist of cIAP and XIAP and leads to degradation of cIAP-1. Treatment of DU145 Sh11.02 cells with 0.1, 1 or 10 μM AT-IAP for 4 h significantly reduced cIAP-1 protein expression (Figure 5A). Addition of TNF-α or monotherapy with AT-IAP had no effect on the cell viability of PTEN-deficient DU145 Sh11.02 cells as determined by MTT assay. However, concurrent administration of TNF-α and AT-IAP reduced cell viability by 44.2 ± 6.5% (*p* < 0.05; Figure 5B). Annexin V-detection by flow cytometry (72 h) and quantitative caspase 3/7 assays (24 h) confirmed that the combination of 10 ng/ml rhTNF-α and 1 μM AT-IAP enhanced the promotion of apoptosis relative to control. Combination therapy with rhTNF-α and AT-IAP increased the Annexin-V-positive cell population by 17.8 ± 4.02% over controls, and induced a 4.7 ± 0.26 fold increase in caspase 3/7 activity (*p* < 0.01; Figure 5C and 5D). Consistent with their inability to significantly impact cell viability in MTT assays, administration of rhTNF-α or AT-IAP alone did not significantly increase apoptosis induction. Similar results were obtained for all these experiments in PTEN-expressing DU145 NT01 cells (Supplementary Figure 4).

### AT-IAP increases radiosensitivity of CaP cells following THP-1 co-culture via modulation of apoptosis

**Figure 6:**
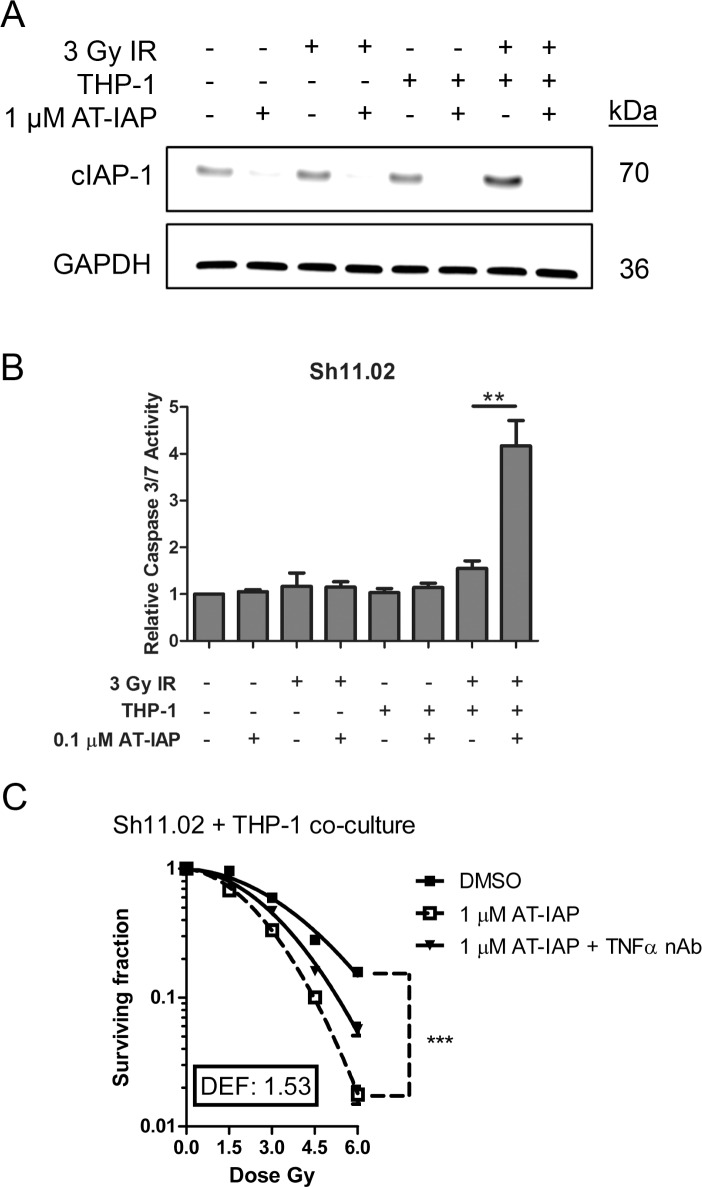
Assessment of the radiosensitizing potential of AT-IAP in Sh11.02 cells (**A**) Immunoblot showing cIAP-1 expression in Sh11.02 cells following THP-1 co-culture, 3 Gy IR and treatment with 0.1 μM AT-IAP. Equal protein loading was confirmed by re-probing for GAPDH. (**B**) Bar graph showing caspase 3/7 activity in Sh11.02 cells following THP-1 co-culture, 3 Gy IR and treatment with 0.1 μM AT-IAP for 6 h. (**C**) Clonogenic survival curve showing the radiosensitizing potential of AT-IAP on Sh11.02 cells with THP-1 co-culture. Data showing addition of a TNF-α neutralizing antibody in this system is also presented. Data shown is the mean plus or minus standard error of the mean value, calculated from a minimum of three independent experiments. Statistically significant differences were determined by performing a two-tailed Students *t*-test or two-way ANOVA for clonogenic assays (*p < 0.05; **p < 0.01; ***p < 0.001).

The ability of AT-IAP to down-regulate cIAP-1 expression in DU145 Sh11.02 cells, cultured in the presence of macrophages and exposed to IR, was assessed by immunoblotting assays. Treatment with 1 μM AT-IAP for 4 h was sufficient to reverse 3 Gy IR and microenvironment-driven, THP-1 mediated increases in cIAP-1 expression (Figure 6A). The addition of AT-IAP and the potent down-regulation of cIAP-1 was sufficient in elevating caspase 3/7 activity, in the presence of the radiation-induced TNF-α release within the co-culture system (Figure 6B). Furthermore, addition of AT-IAP increased the radiation sensitivity of DU145 Sh11.02 cells in the presence of THP-1 cells, resulting in a calculated DEF of 1.53 (*p* < 0.001; Figure 6C). Interestingly, this effect was only partially knocked back following addition of a TNF-α neutralizing antibody (*p* < 0.001), suggesting that other monocyte-derived secreted factors may by influencing CaP cell radiosensitivity. AT-IAP also enhanced the radiation sensitivity of PTEN-expressing DU145 NT01 cells (Supplementary Figure 4E).

## DISCUSSION

Loss of the tumor suppressor PTEN is prevalent in men presenting with organ-confined prostate cancer and has prognostic value towards predicting poor response to radiotherapy [4]. We have previously shown that impairment of PTEN function correlates with an elevated expression of the CXC-chemokine CXCL8 in *in vitro* models and its orthologous chemokine KC in the prostate epithelium of PTEN^+/−^ mice [5]. In addition to the definition of several autocrine functions, CXCL8 has the potential to register profound effects on the tumor microenvironment, predominantly through angiogenesis or driving chemotactic migration of immune cells [24]. In this study, *in vitro* assays reaffirm that one impact of tumor-derived CXCL8 from PTEN-deficient tumor cells is to enhance chemotactic migration of macrophages. Furthermore, we have shown that radiation-induced macrophage infiltration may also be inhibited by blockade of CXCL8 signaling. Through immunohistochemical analysis of human prostate cancer tissue, we have demonstrated that loss of PTEN-expression correlates with a greater incidence of macrophage infiltration. Previous studies have discussed the potential relevance of TAMs in patients treated by RP [25, 26]. Using optimized *in vitro* systems, we have shown that co-culture with macrophage representative THP-1 cells increased the radio-resistance of CaP cells, suggesting that the higher level of macrophage infiltration in PTEN-deficient tumors may underpin their adverse prognosis with respect to radiotherapy (4).

Founded on the detection of macrophage enrichment in PTEN-deficient prostate tumors, we evaluated how the cellular compartments, studied in isolation and in co-culture responded to clinically-relevant doses of IR. Cytokine arrays were conducted to determine how exposure to radiation may affect the secretion of these factors. Our data confirms that prostate epithelial cells and macrophages are both a rich source of constitutive TNF-α secretion *in vitro*. Profiling of micro-dissected tumor epithelial tissue also confirms equivalent expression of this inflammatory marker in both PTEN-expressing and PTEN-deficient contexts. Experimental data indicated that exposure to IR further induced TNF-α secretion from THP-1 cells but had no effect on further potentiating the endogenous levels of secreted TNF-α from PTEN-deficient Sh11.02 cells. Extrapolating to the treatment of patients, this would suggest that the altered constitution of the microenvironment and especially, the density of TAM infiltration in PTEN-deficient tumors would be an important determinant in dictating the impact of radiation in increasing the local concentration of bioactive TNF-α present within tumor foci.

TNF-α is known to be an activator of either apoptotic or pro-survival signaling [12]. Switching between survival- and death-promoting signaling is principally dependent on its ability to activate NFκB transcription [27, 28]. Consistent with the resistance of CaP cells to clinically relevant doses of TNF-α, this cytokine was shown to increase NFκB signaling and increase expression of c-FLIP, cIAP-1 and Bcl-2, three known NFκB-regulated anti-apoptotic targets in prostate cancer cells (30, 31). The activation of NFκB was further increased following radiation and constitution of macrophage-enriched tumor microenvironment, but was inhibited by blockade of TNF-α signaling. Expression of c-FLIP and cIAP-1 was further increased in irradiated CaP cells in the presence of THP-1 cells, suggesting that the inhibition of caspase-dependent apoptosis may be one principal underlying mechanism of resistance afforded by this cytokine.

The mode-of-action of cIAP-1 is well understood. This protein is recruited to death receptors following ligand binding and is responsible for resultant NFκB activation by promoting the polyubiquitination of RIP-1 kinase (RIP1) [29]. Inhibition of cIAP-1 is therefore viewed as an attractive and potential amenable target to direct the downstream fate stemming from cytokine signaling, switching from primarily a cell survival response to a pro-apoptotic cell death phenotype. Several Smac-mimetics, have demonstrated efficacy in a range of cancer types [30, 31], including prior demonstration that they can enhance radiotherapy sensitivity in CaP xenograft models [32]. In our hands, treatment with a novel IAP antagonist, AT-IAP, ablated the expression of cIAP-1 in DU145 Sh11.02 cells, consistent with a proteasome-dependent degradation of the protein. When combined with exogenous TNF-α, AT-IAP reversed the resistance of CaP cells to this cytokine, and significantly reduced cell viability by promoting apoptosis *in vitro*. Moreover, AT-IAP retained the capacity to augment apoptosis levels in irradiated CaP cells in the co-culture model, consistent with this agent working in synergy with radiation-induced macrophage-derived TNF-α. Moreover, in clonogenic survival assays, the administration of AT-IAP reversed the resistance afforded by macrophages, instead increasing the sensitivity of PTEN-deficient Sh11.02 cells to clinically-relevant doses of external-beam radiotherapy.

Macrophage-derived TNF-α signaling has previously been implicated in promoting radio-resistance in *in vivo* models of melanoma [7]. We have detected the enrichment of macrophages in PTEN-deficient prostate tumors, and extrapolating to use of co-culture models, we have demonstrated the relevance of macrophages and TNF-α signaling in contributing to the radio-resistance of CaP cells. Moreover, our experimental data reveals that use of an IAP antagonist confers increased radio-sensitivity of PTEN-deficient cancer cells, exploiting the inflammatory potential of the macrophage-enriched microenvironment.

## CONCLUSION

PTEN-deficient tumors exhibit an elevated macrophage infiltration underpinning increased macrophage-promoted TNF-α signaling that promotes survival of CaP cells through up-regulation of anti-apoptotic proteins including cIAP-1. Treatment with a novel cIAP-1 antagonist, AT-IAP, sensitizes malignant prostate cells to radiotherapy, suggesting that Smac mimetics may be a relevant therapeutic strategy for enhancing the effect of ionizing radiation in patients presenting with PTEN-deficient CaP.

## MATERIALS AND METHODS

### Reagents

Recombinant human TNF-α was purchased from Alomone Labs (Jerusalem, Israel) and cells were treated at a final concentration of 10 ng/ml. Human TNF-α neutralizing antibody was obtained from Cell Signaling (Beverly, MA, USA) and cells were treated at a final concentration of 10 ng/ml. Recombinant human CXCL8 was purchased from Peprotech (London, UK) and cells were treated at a final concentration of 3 nM. Human CXCL8 neutralizing antibody was obtained from R & D Systems (Abingdon, UK). AT-IAP was supplied by Astex Pharmaceuticals (Cambridge, UK) and reconstituted in DMSO before treating at a final concentration of 1 μM unless otherwise stated.

### Cell line and cell culture

Authenticated DU145 prostate cancer cells were obtained from American Type Culture Collection (ATCC); PTEN expression was depleted and cells cultured as previously described [5]. Human macrophage-like THP-1 cells were maintained in RPMI medium supplemented with 10% FCS and 0.05 mM β-mercaptoethanol. In all co-culture experiments, THP-1 cells were seeded at a density which represented 20% of the prostate cancer cell population. Cells were regularly tested to ensure they were free of mycoplasma contamination.

### DNA and RNA extraction from FFPE clinical samples

Twenty-eight Gleason 7 grade prostate tumour FFPE blocks were obtained from the Northern Ireland Biobank (NIB13–0074). Sequential 4 μm sections of each case were cut and placed on glass slides (a total of 20 μm). The tumour regions were then macro-dissected into sterile 1.5 ml Eppendorf tubes. The RNA was extracted using the Qiagen RNAeasy kit according to manufacturer's instructions. Genomic DNA was extracted using the Promega Genomic DNA extraction kit. All standard procedures were taken according to the manufacturer's protocol. Extracted DNA and RNA was quantified and assessed for their quality using the Agilent Bioanalyser chips.

### Whole genome gene expression (WG-DASL)

Whole genome gene expression analysis was performed using the Illumina (San Diego, CA, USA) WG-DASL assay according to manufacturer's protocol. Briefly, 100 ng of FFPE RNA was converted to cDNA by the WG-DASL assay using biotinylated-tagged random nonamer and oligo (dT) primers. The biontinylated cDNA was then mounted onto a streptavidin-coated support and further extended and ligated by gene-specific oligonucleotides (DAP). Subsequently, PCR amplification was performed. The resulting PCR products were eluted and hybridized to the Illumina Human-Ref v3.0 Beadchip and scanned with the Illumina iScan Reader. The image intensity values from the microarray images generated were then analysed by the GenomeStudio Gene Expression Module (Illumina, San Diego, CA, USA) software. The processed methylation values were subsequently used for further analysis in this study.

### Whole genome methylation

Whole genome methylation analysis was performed using the Illumina Infinium HD (San Diego, CA, USA) assay according to manufacturer's protocol. Briefly, 1000 ng of genomic DNA extracted from the FFPE samples was firstly treated with sodium bisuphite to convert unmethylated cytosine to uracil. The bisulphite treated DNA was denatured isothermally and amplified overnight. After amplification, the post-amplified DNA was fragmented using a proprietary enzymatic process and precipitated using isopropanol. The precipitated DNA was then collected by centrifugation and re-suspended in a hybridization buffer. The hybridized product was then hybridized onto the Infinium 450K Beadchip. The loaded chip underwent further extension and staining steps. Subsequently, the Illumina iScan reader was used to derive image intensity values off the stained chip from the high-resolution scans of the chip. The image intensity values was processed and normalized by the GenomeStudio Gene Expression Module (Illumina, San Diego, CA, USA). The processed methylation values were subsequently used for further analysis in this study.

### Immunohistochemistry

Tissue microarrays consisting of Gleason 6 (*N* = 15) and Gleason 7 (*N* = 55) prostate tumors were obtained from the Northern Ireland Biobank. Consecutive TMA paraffin sections of 4 μm thickness were cut and placed onto silanated slides for immunohistochemical detection of PTEN and CD68 using a Bond-Max autostainer (Leica Biosystems, Newcastle, UK). Standard processing steps were performed according to manufacturer's instructions. Briefly, heat-induced antigen retrieval with epitope retrieval ER1 solution (Leica Biosystems) was performed for 20 min prior to incubation with primary antibody. Slides were incubated with PTEN (Cascade Bioscience, clone 6H2.1, dilution 1:400) or CD68 (BD Pharmingen, clone KP1, dilution 1:250). After incubation, slides were washed with Bond washing buffer (Leica Biosystems) and incubated with secondary antibody (Bond Polymer Refine kit, Leica Biosystems). Subsequently chromogenic detection was achieved by incubation with 3, 30-diaminobenzidine (DAB) followed by Bond DAB enhancer (Leica Biosystems). All slides were counterstained with haematoxylin and dehydrated through ascending ethanol to xylene before mounting. Expression of PTEN was scored either as positive (retention of cytoplasmic staining) or negative (lack of staining). Expression of CD68 was scored based on their staining pattern (either dense or sparse) of the macrophages.

### Western blotting analysis

Whole cell lysates were prepared, resolved and blotted as previously described [5]. Membranes were probed with the following primary antibodies: anti-PTEN (Cell Signaling, Beverly, MA, USA); anti-c-FLIP (Enzo Life Sciences, Exeter, UK); anti-cIAP-1 (Santa Cruz, Heidelberg, Germany); anti-Bcl-2 (Cell Signaling, Beverly, MA, USA); anti-TNFR-1 (Santa Cruz, Heidelberg, Germany); and anti-Caspase-8 (Millipore, Billerica, MA, USA) at 4°C overnight. Membranes were washed three times with 1X PBS + 0.05% Tween-20 before being incubated with the appropriate horseradish peroxidase (HRP)-tagged secondary antibody (GE Healthcare, UK). Immunolabelled proteins were detected using the Luminata Crescendo substrate (Millipore, Billerica, MA, USA). Membranes were reprobed with GAPDH primary antibody (ABD Serotec, UK) to ensure equal loading.

### Radiation treatments

Cells were exposed to 225 kVp x-rays using the self-contained X-RAD 225 irradiation system (Precision X-Ray Inc., Connecticut, USA). All experiments were performed at a dose rate of 0.52 Gy min^−1^.

### Chemotaxis assay

Analysis of THP-1 chemotaxis was performed using the 5 μm pore size CytoSelect^™^ 96-Well Cell Migration Assay kit (Cell Biolabs) according to manufacturer's instructions. For conditioned media experiments, prostate cancer cells were treated with a 3 Gy dose of IR and incubated at 37°C for 6 h prior to collecting media.

### Clonogenic assays

Prostate cancer cells were plated into 6-well plates at a final density determined by the dose of IR to be received. At the same time, THP-1 cells were added directly to each well intended for co-culture at a density that was 20% of the total CaP cell number. Cells were left to incubate at 37°C overnight before being treated accordingly the following morning. For AT-IAP treatments, cells were allowed to incubate for 1 h prior to being irradiated. Following IR, plates were returned to the incubator for 10–14 days until colonies in the control plate had reached a size of 50 cells or more. THP-1 cells were removed by rinsing the wells with 1X PBS prior to fixation and staining by 0.4% crystal violet. Surviving fractions (SF) were calculated relative to non-irradiated cells and fitted using a linear quadratic function (S = exp(−αD -βD^2^)) using least-squares regression (Prism 5.0; GraphPad Software, CA). Area under-the-curve (AUC) representing the mean inactivation dose (MID) was obtained and dose enhancement factor (DEF) calculated by dividing the MID of the control by that of the treated group.

### ELISA

Secreted CXCL8 and TNF-α in culture media were analyzed using commercially available ELISA kits (Pelikine, Beckman Coulter, High Wycombe, UK and Biolegend, London, UK respectively) as previously described [5].

### Luciferase reporter assay

NFκB luciferase assays were performed using the NFκB-LUC-PGL4 plasmid (Promega, Southampton, UK) as previously described [20].

### Detection of apoptosis

Cells were seeded at a density of 1 × 10^5^ into 6-well plates and left to adhere overnight. Cells were then treated with either rhTNF-α, AT-IAP or both and returned to the incubator for 72 h. Whole culture medium was collected and pooled with the corresponding trypsinized cells before being pelleted via centrifugation at 1000 rpm. Cell pellets were resuspended in 100 μl of binding buffer and 5 μl of Annexin V antibody (Life Technologies, Paisley, UK) was added to each sample alongside 5 μl of propidium iodide (PI) stain (50 μg/ml). Samples were incubated in darkness for 15 min before adding 320 μl of binding buffer and analyzing on the EPICS XL flow cytometer (Beckman Coulter, Buckinghamshire, UK). Caspase 3/7 activity assays were performed according to manufacturer's instructions (Promega, UK).

### MTT assay

Cells were treated with rhTNF-α, AT-IAP or both for 72 h and cell viability was then assessed as previously described [21].

### Statistical analysis

Statistically significant differences between means were determined using a two-tailed Students *t*-test. Statistical significance between different treatment groups following clonogenic survival assay were determined by performing a two-way ANOVA. All significant statistical differences were defined as *P* < 0.05 (*), *P* < 0.01 (**) and *P* < 0.001 (***).

## SUPPLEMENTARY MATERIALS


