# Supplementary material for: PTEN deficiency promotes macrophage infiltration and hypersensitivity of prostate cancer to IAP antagonist/radiation combination therapy
Source: Oncotarget. 2016 Jan 20;7(7):7885–98. doi: 10.18632/oncotarget.6955 (PMC4884961; doi:10.18632/oncotarget.6955)
Supplement: Supplementary file 1 [file oncotarget-07-7885-s001.pdf]

PTEN deficiency promotes macrophage infiltration and hypersensitivity of prostate cancer to IAP antagonist/radiation combination therapy

Supplementary Materials

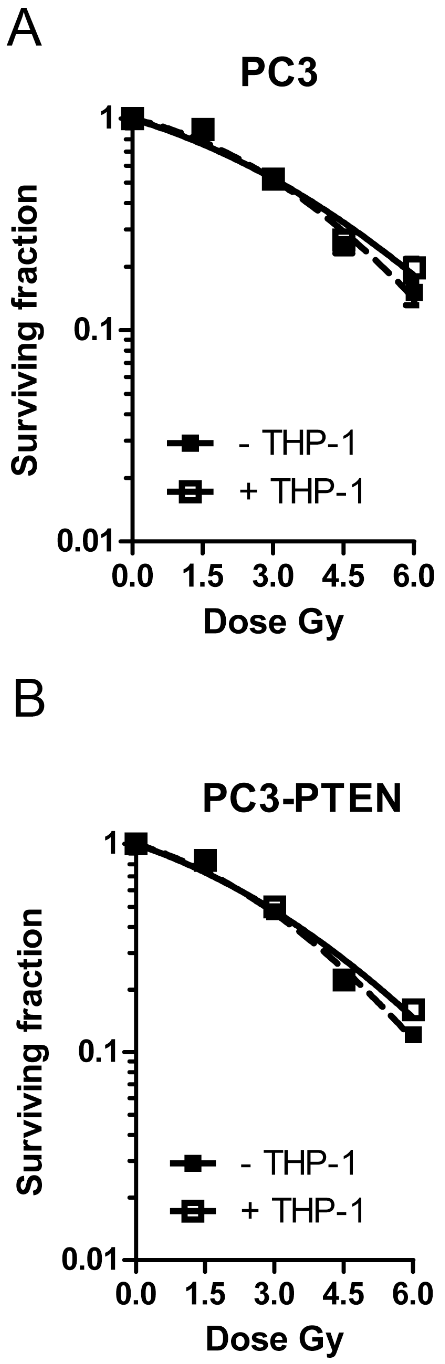

Supplementary Figure S1: Impact of THP-1 co-culture on PC3 cell radiosensitivity. (**A**) Clonogenic survival curves showing the radiation response of PTEN-null PC3 cells in the presence or absence of THP-1 co-culture. (**B**) Clonogenic survival curves showing the radiation response of PC3 cells with reconstituted PTEN expression in the presence or absence of THP-1 co-culture.

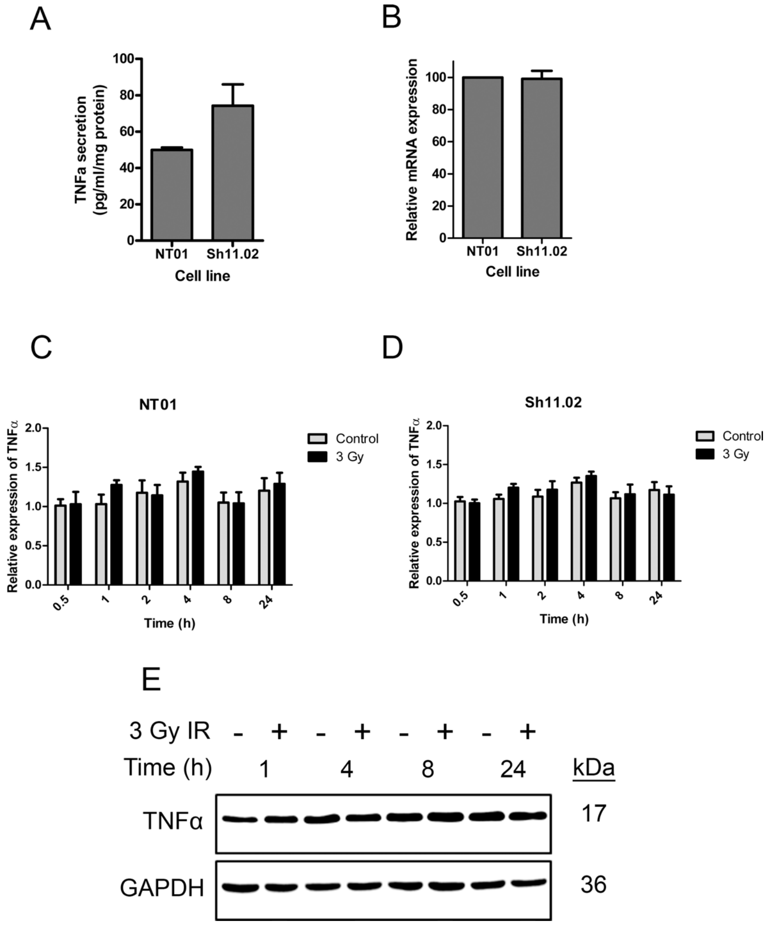

Supplementary Figure S2: TNF-α expression and secretion in PTEN-modulated DU145 cells. (**A**) Bar graph illustrating basal secretion levels of TNF-α in DU145 NT01 and DU145 Sh11.02 cells. (**B**) Bar graph illustrating qRT-PCR analysis of basal TNF-α mRNA expression in DU145 clonal populations. (**C**) Bar graph showing the effect of 3 Gy IR on TNF-α mRNA expression in PTEN-expressing NT01 cells. (**D**) Bar graph showing the effect of 3 Gy IR on TNF-α mRNA expression in PTEN-depleted Sh11.02 cells. (**E**) Immunoblot showing TNF-α protein expression in Sh11.02 cells following treatment with a 3 Gy dose of IR. Data shown is the mean plus or minus standard error of the mean value, calculated from a minimum of three independent experiments.

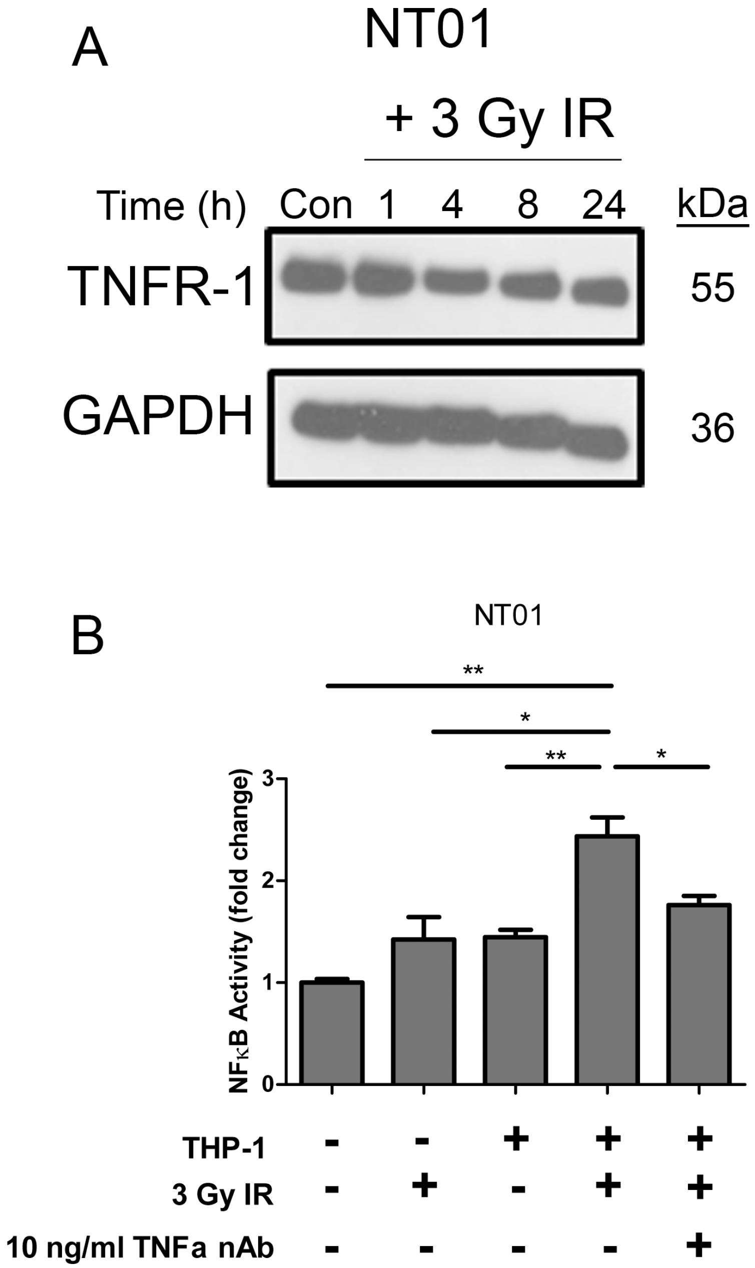

Supplementary Figure S3: Impact of ionizing radiation and THP-1 co-culture on the NT01 TNF-α/NFκB signaling axis. (**A**) Immunoblot showing the impact of a single 3 Gy dose of IR on expression of TNFR-1 in NT01 cells. (**B**) Bar graph illustrating luciferase reporter assay analysis of NFκB activity in NT01 cells. Different experimental conditions included THP-1 co-culture, exposure to 3 Gy IR and treatment with 10 ng/ml TNF-α neutralizing antibody. Data shown is the mean plus or minus standard error of the mean value, calculated from a minimum of three independent experiments. Statistically significant differences in luciferase activity were determined by performing a two-tailed Students *t*-test.

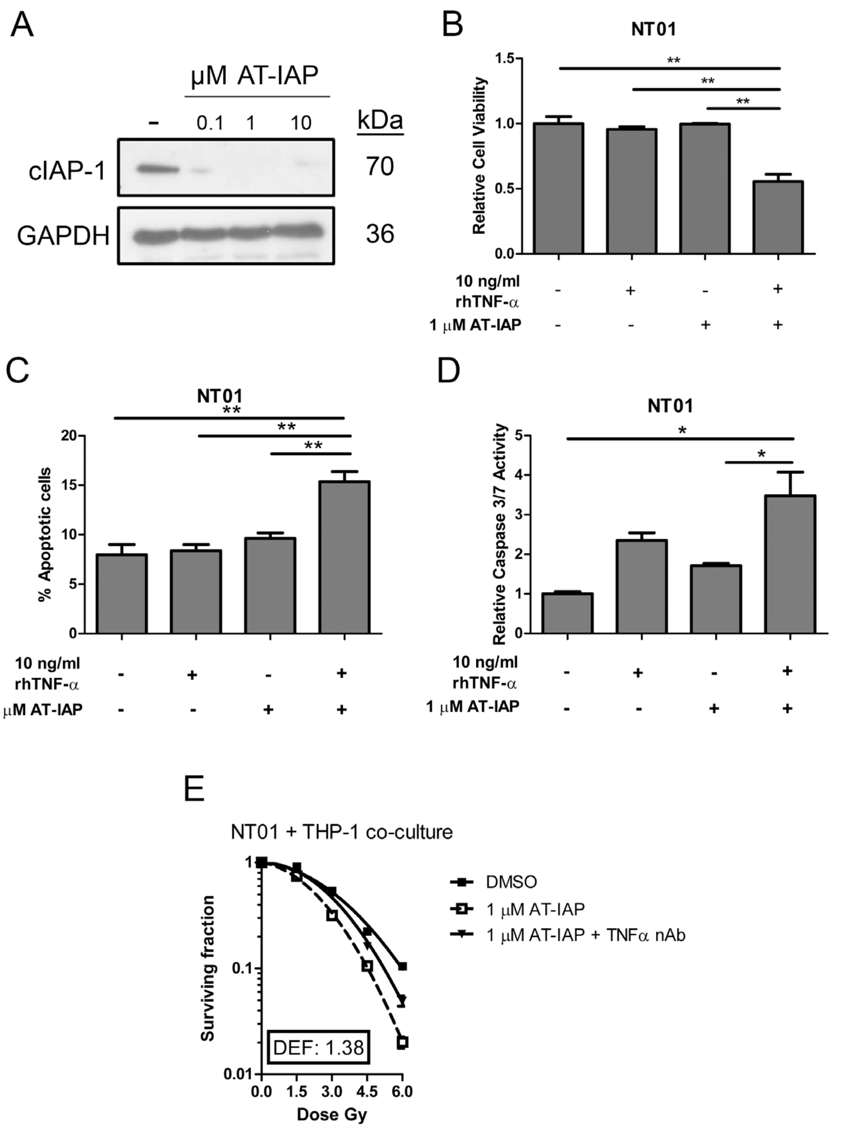

Supplementary Figure S4: Impact of AT-IAP on cell viability and the radiation response of NT01 cells. (**A**) Immunoblot showing cIAP-1 expression in NT01 cells following treatment with 0.1, 1 or 10 µM AT-IAP. Cells were also treated with 10 ng/ml TNF-α to ensure pathway activation. Equal protein loading was confirmed by re-probing for GAPDH. (**B**) Bar graph presenting MTT assay analysis of NT01 cells 72 h following treatment with 10 ng/ml TNF-α, 1 µM AT-IAP, or a combination of both. (**C**) Bar graph illustrating flow cytometry data following Annexin V/PI staining of NT01 cells. Different treatment conditions were similar to those mentioned above. (**D**) Bar graph showing caspase 3/7 activity of NT01 cells following treatment with rhTNF-α, AT-IAP or both in combination for 24 h. (**E**) Clonogenic survival curve showing the radiosensitizing potential of AT-IAP on NT01 cells with THP-1 co-culture. Data shown is the mean plus or minus standard error of the mean value, calculated from a minimum of three independent experiments. Statistically significant differences were determined by performing a two-tailed Students *t*-test or two-way ANOVA for clonogenic assays.

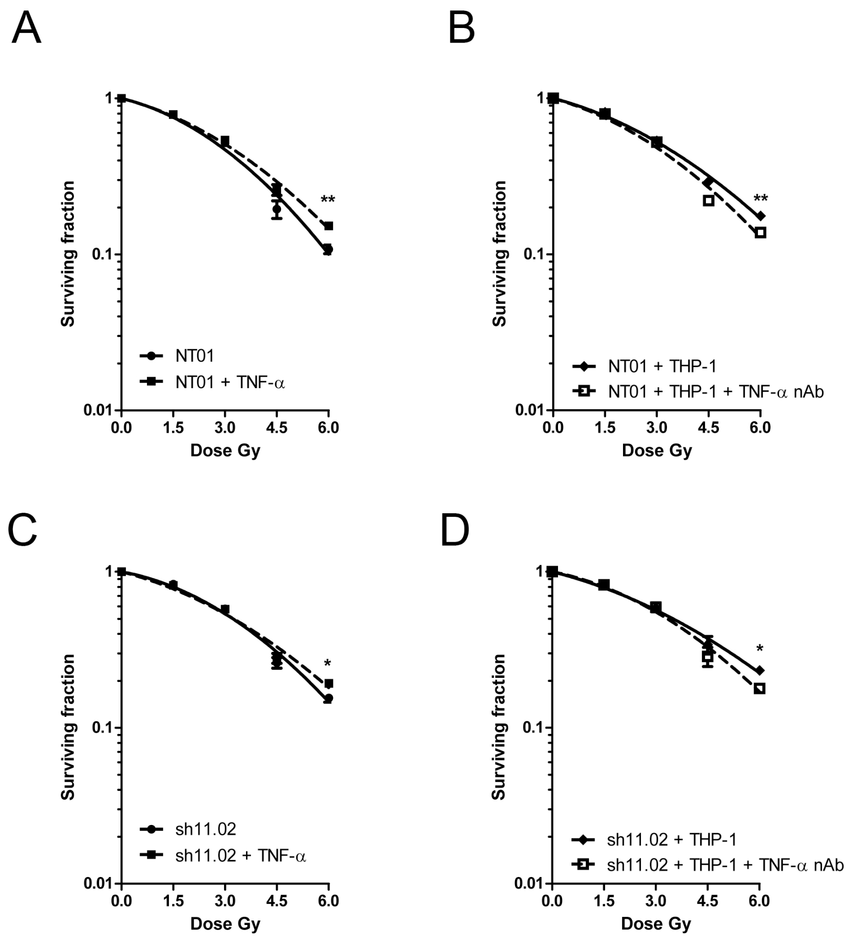

Supplementary Figure S5: Effect of rhTNF-α on the radiation response of DU145 isogenic cell lines. (**A**) Clonogenic survival curves illustrating the effect of 10 ng/ml recombinant TNF-α on the radiation response of NT01 cells. (**B**) Clonogenic survival curves showing the effect of 10 ng/ml TNF-α neutralizing antibody on the radiation response of NT01 cells following THP-1 co-culture. (**C**) Clonogenic survival curves illustrating the effect of 10 ng/ml recombinant TNF-α on the radiation response of Sh11.02 cells. (**D**) Clonogenic survival curves showing the effect of 10 ng/ml TNF-α neutralizing antibody on the radiation response of Sh11.02 cells following THP-1 co-culture. Data shown is the mean plus or minus standard error of the mean value, calculated from a minimum of three independent experiments. Statistically significant differences at individual dose points were determined by performing a two-tailed Students *t*-test.
